# An illustrated key to and diagnoses of the species of Histeridae (Coleoptera) associated with decaying carcasses in Argentina

**DOI:** 10.3897/zookeys.261.4226

**Published:** 2013-01-24

**Authors:** Fernando H. Aballay, Gerardo Arriagada, Gustavo E. Flores, Néstor D. Centeno

**Affiliations:** 1 Laboratorio de Entomología, Instituto Argentino de Investigaciones de las Zonas Áridas (IADIZA, CCT CONICET Mendoza), Casilla de correo 507, 5500 Mendoza, Argentina; 2 Sociedad Chilena de Entomologia; 3 Laboratorio de Entomología Aplicada y Forense, Universidad Nacional de Quilmes, Roque Sáenz peña 180, B1876BXD, Bernal, Buenos Aires, Argentina

**Keywords:** Key, Histeridae, Saprininae, forensic, carcasses, Argentina

## Abstract

A key to 16 histerid species associated with decaying carcasses in Argentina is presented, including diagnoses and habitus photographs for these species. This article provides a table of all species associated with carcasses, detailing the substrate from which they were collected and geographical distribution by province. All 16 Histeridae species registered are grouped into three subfamilies: Saprininae (twelve species of *Euspilotus* Lewis and one species of *Xerosaprinus* Wenzel), Histerinae (one species of *Hololepta* Paykull and one species of *Phelister* Marseul) and Dendrophilinae (one species of *Carcinops* Marseul). Two species are new records for Argentina: *Phelister rufinotus* Marseuland *Carcinops troglodytes* (Paykull). A discussion is presented on the potential forensic importance of some species collected on human and pig carcasses.

## Introduction

Coleoptera is one of the major orders of insects represented on carcasses and its forensic importance has been frequently documented (Benecke 1988, Kulshrestha and Satpathy 2001, Schroeder et al. 2002). Among the most important families mentioned in the literature are: Dermestidae, Cleridae, Histeridae, Staphylinidae, Nitidulidae, Scarabaeidae, Tenebrionidae, and Trogidae (Mise et al. 2007, Özdemir and Set 2009, Almeida and Mise 2009, Battán Horenstein and Linhares 2011). Members of these families are associated with carcasses due to different trophic roles of adults or their immature stages, which can feed on cadaveric tissues (necrophagous) or on other insects in the body, such as larvae of Diptera or other Coleoptera species (necrophilous).

Histeridae comprises 4252 species and 391 genera worldwide, grouped in 11 subfamilies (Mazur 2011), with 139 genera and 1047 species in the Neotropical region (Almeida and Mise 2009). They are mostly predators of soft-bodied insect larvae and eggs, particularly of cyclorraphan Diptera, whose larvae develop on carcasses and dung of large mammals. The odoriferous products of microbial degradation attract both flies and histerids via olfaction (Kovarik and Catherino 2001).

Due to the fact that the Diptera colonize the body from the beginning of the decomposition process (Goff 1993), they have been the group most used for estimating Post Mortem Interval (PMI) within short periods after death. However, they have little usefulness after several weeks or months, when the body is in advanced stages of decomposition. Although histerids are less abundant than flies in the scavenger community, they complete its life cycle in the body (Aballay pers. obs.). Thus, histerids may be helpful when a long time has elapsed since death. Adult histerids reach their highest abundance in intermediate stages of decomposition such as Active and Advanced Decay (Özdemir and Set 2009) and can cause a remarkable decrease in the number of immature stages of Diptera: Calliphoridae (Nuorteva 1970).

Histerid adults have been frequently mentioned in forensic studies on decomposing pig carcasses (Wolff et al. 2001, Centeno et al. 2002, Aballay et al. 2008, Özdemir and Sert 2009, Battán Horenstein and Linhares 2011, Battán Horenstein et al. 2012, Aballay 2012) and on human corpses (Arnaldos et al. 2005, Mariani et al. 2010, Aballay obs. pers.). The correct identification of insects and knowledge of their life history as well as the duration of each stage of development leads to accurately establishing the PMI (Turchetto and Vanin 2004). In South America, the usefulness of histerids as PMI indicators has not been established due to the absence of taxonomic keys that allow their determination, as well as minimal documentation of detailed life histories. In previous forensic studies in the continent, histerids were identified to family level (Mariani et al. 2010), most to generic level (Carvalho et al. 2000, Wolff et al. 2001, Mise et al. 2007, Segura et al. 2009, Battán Horenstein and Linhares 2011, Battán Horenstein et al. 2012) and a few to species level (Centeno et al. 2002, Oliva and Ravioli 2004, Aballay et al. 2008, Mise et al. 2010). A key to the main families of South American Coleoptera of forensic importance was recently published (Almeida and Mise 2009), which includes histerids mentioned for some South American countries. In this key, only six genera and two species were determined for Histeridae, and it does not include most of the species collected in Argentina in decomposition assays (Aballay et al. 2008). For these reasons, it is necessary to have a tool that allows determination of the necrophilous species of Histeridae.

The objective of this paper is to provide an illustrated key to the histerid species associated with decaying carcasses in Argentina to achieve their correct identification. Additionally, diagnoses for these species are presented.

## Material and methods

A total of 7070 specimens were collected mostly during forensic studies on decomposing pig carcasses because it is the preferred animal model for forensic entomological studies (Goff 1993). These decomposition experiments were conducted in three Argentinean provinces with arid conditions: Mendoza, San Juan and Catamarca. Histerids were collected during the entire decomposition process on 16 pig carcasses. In Mendoza, the study was carried out at the campus of Instituto Argentino de Investigaciones de las Zonas Áridas, CCT CONICET-Mendoza (32°53'53.3"S, 68°52'26.2"W, 850 m altitude) collecting histerids on 12 pig carcasses, during the four seasons of the year. In San Juan, histerids were collected on two decomposing pig carcasses in summer at the campus of Facultad de Ciencias Exactas Físicas y Naturales, Universidad Nacional de San Juan (31°32'34.1"S, 68°34'38.2"W, 673 m altitude). In Catamarca, histerids were collected on two decomposing pig carcasses during spring in Antofagasta de la Sierra (26°01'32.3"S, 67°20'36.5"W, 3600 m altitude).

In addition specimens from decomposing pig carcasses were recorded in the provinces of Salta (24°54'40"S, 65°28'16"W, 1379 m altitude) and Jujuy (24°09'54.13"S, 65°18'37.73"W, 1383 m altitude), with mesic conditions. For collecting and conserving specimens the methodology followed was that by Centeno et al. (2002) and Aballay et al. (2008, 2012).

Other Histeridae specimens were obtained using three kinds of collecting procedures, the first was conducted on human corpses at different places in Mendoza province authorized by the Medical Forensic Committee of Mendoza; the second was conducted in field trips in different Argentinean provinces on carcasses of cow (*Bos taurus*), horse (*Equus caballus*), donkey (*Equus asinus*), dog(*Canis familiaris*), snake (not identified), Geoffroy´s cat(*Leopardus geoffroyi*), llama(*Lama glama*), guanaco (*Lama guanicoe*), vicuña (*Vicugna vicugna*), sheep (*Ovis orientalis*), fox (*Lycalopex griseus*), lesser rhea (*Pterocnemia pennata*), rat (*Eligmodontia typus*) all found outdoors; the third type of collection was using traps baited with rotting flesh of chicken, squid and sardine in different provinces of Argentina.

Voucher specimens are deposited in the entomological collections of the Instituto Argentino de Investigaciones de las Zonas Áridas (Mendoza, Argentina) and Museo Nacional de Historia Natural (Santiago, Chile).

**Figure 1.**
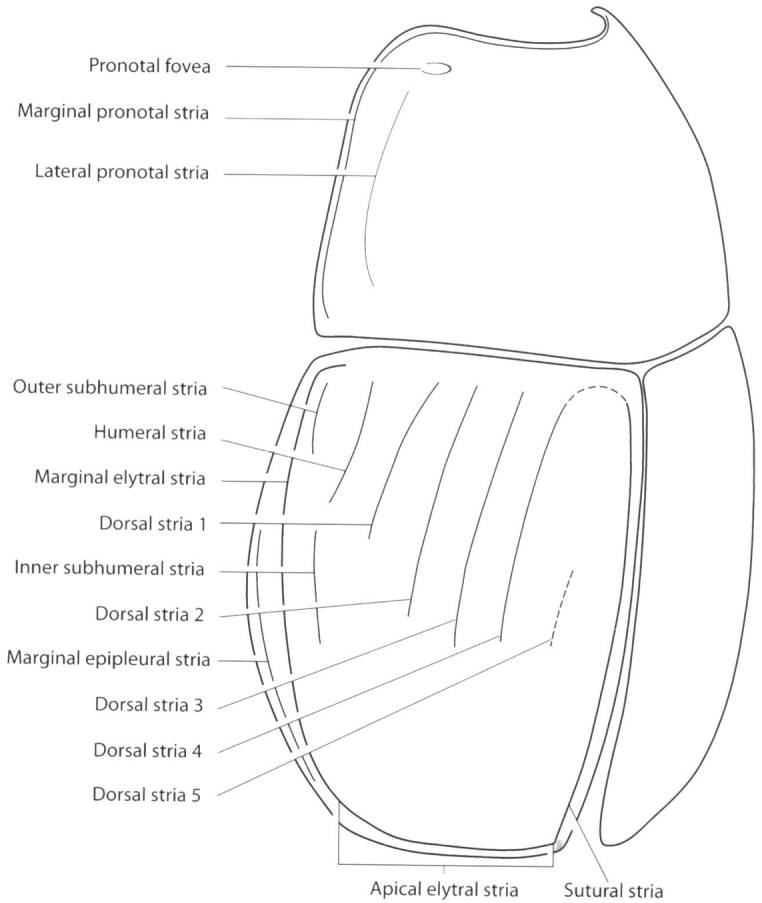
Saprininae, schematic. Pronotum and elytra, oblique lateral view (taken from Lackner 2010).

**Figure 2.**
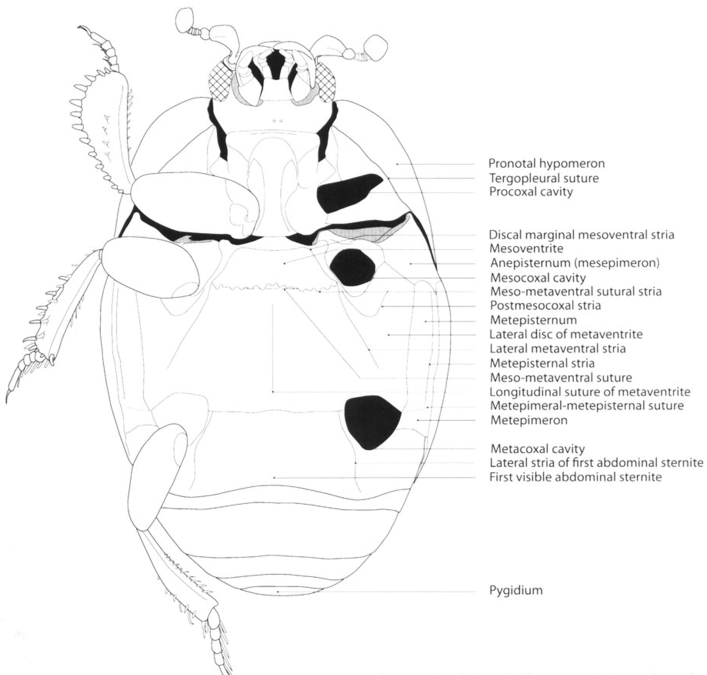
Saprininae, schematic. Habitus, ventral view (taken from Lackner 2010).

**Figures 3–4.**
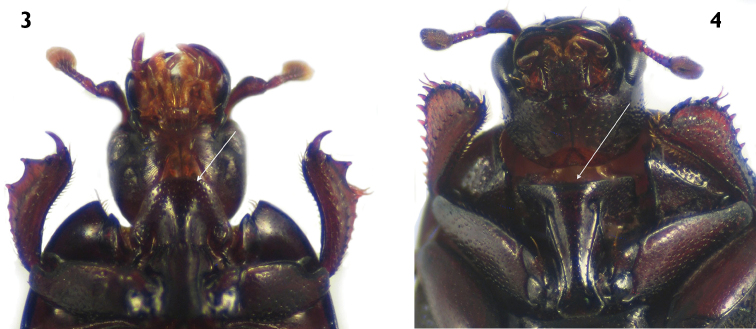
Prosternum in ventral view. **3** *Carcinops (Carcinops) troglodytes* **4** *Euspilotus (Hesperosaprinus) modestus*.

**Figures 5–10.**
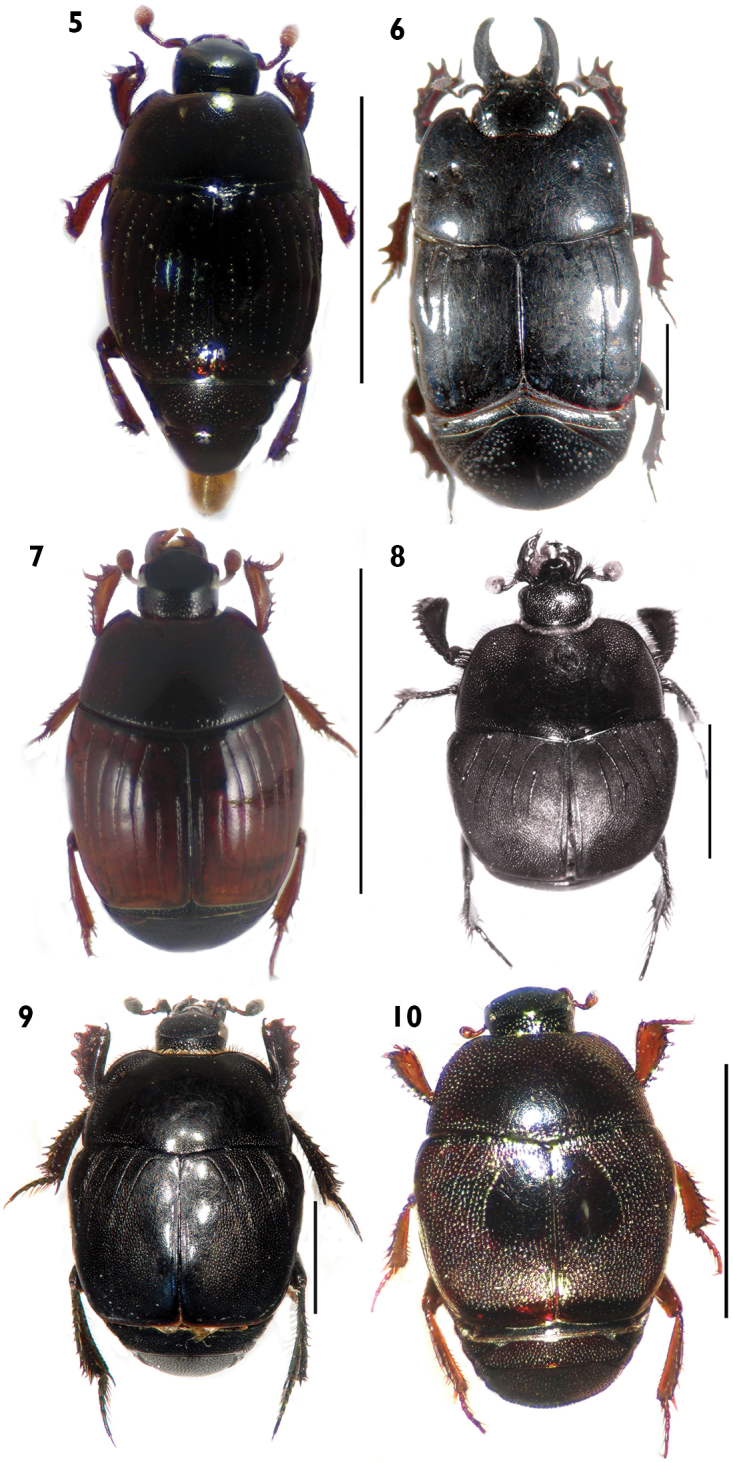
Habitus in dorsal view. **5***Carcinops (Carcinops) troglodytes* **6** *Hololepta (Leionota) reichii*. **7** *Phelister rufinotus* **8** *Euspilotus* (*s. str*.) *lacordairei* **9** *Euspilotus* (s. str.) *patagonicus* **10** *Xerosaprinus (Xerosaprinus) diptychus*. Scale bars: 2 mm. Scale bars: 2 mm.

**Figures 11–12.**
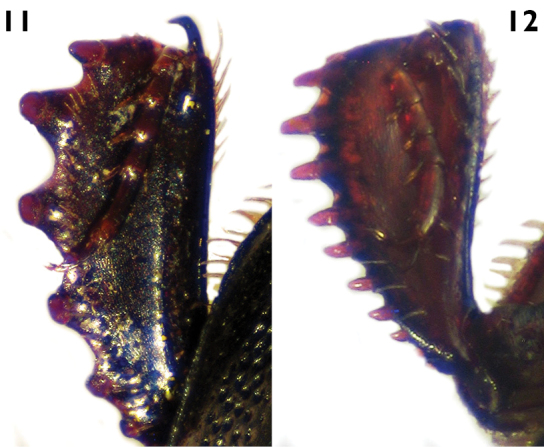
Protibia in dorsal view. **11** *Euspilotus* (s. str.) *patagonicus* **12** *Xerosaprinus (Xerosaprinus) diptychus*.

**Figures 13–18.**
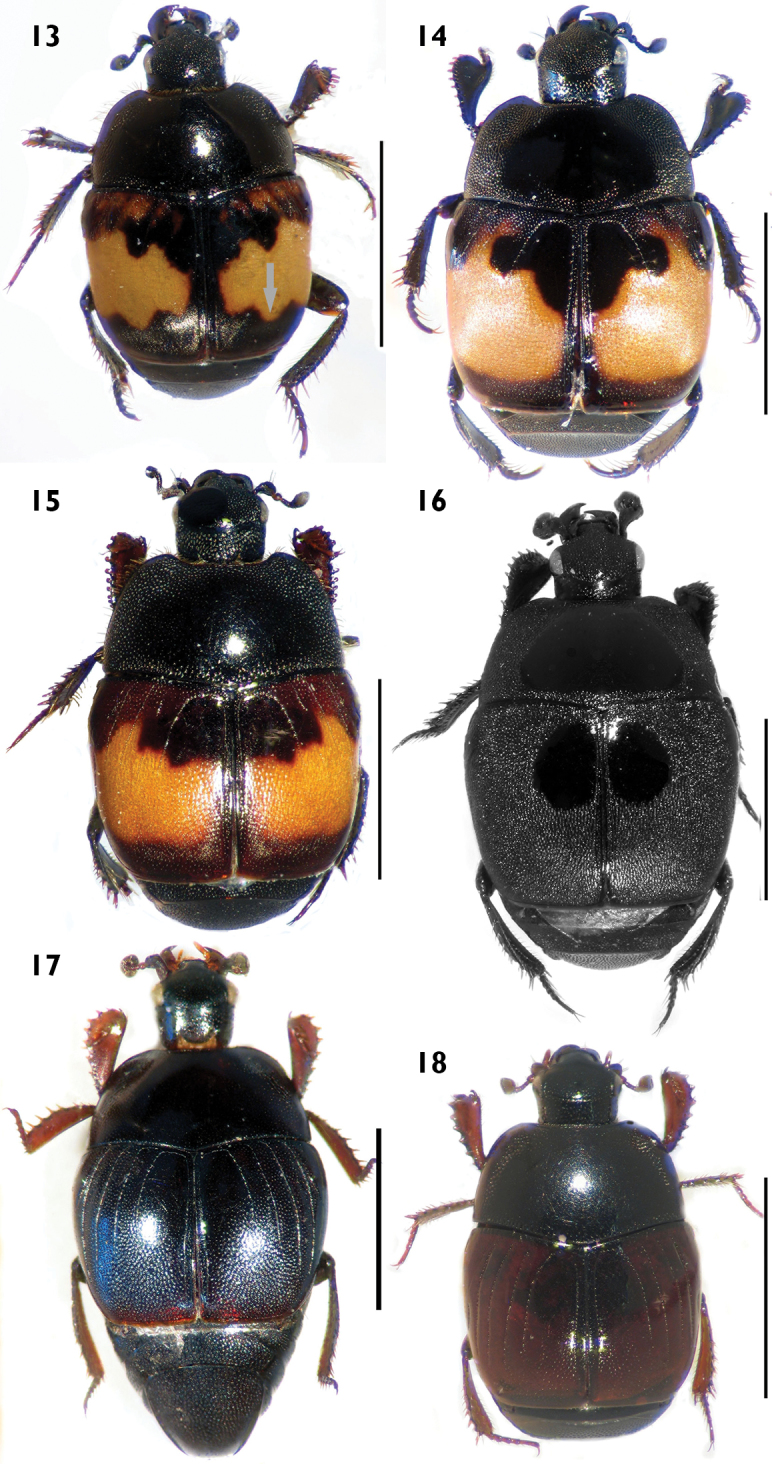
Habitus in dorsal view. **13** *Euspilotus* (s. str.) *richteri* **14** *Euspilotus* (s. str.) *lepidus* **15** *Euspilotus* (s. str.) *ornatus* **16** *Euspilotus (Hesperosaprinus) caesopygus* **17** *Euspilotus (Hesperosaprinus) strobeli* **18** *Euspilotus (Hesperosaprinus) pavidus*. Scale bars: 2 mm.

**Figures 19–22.**
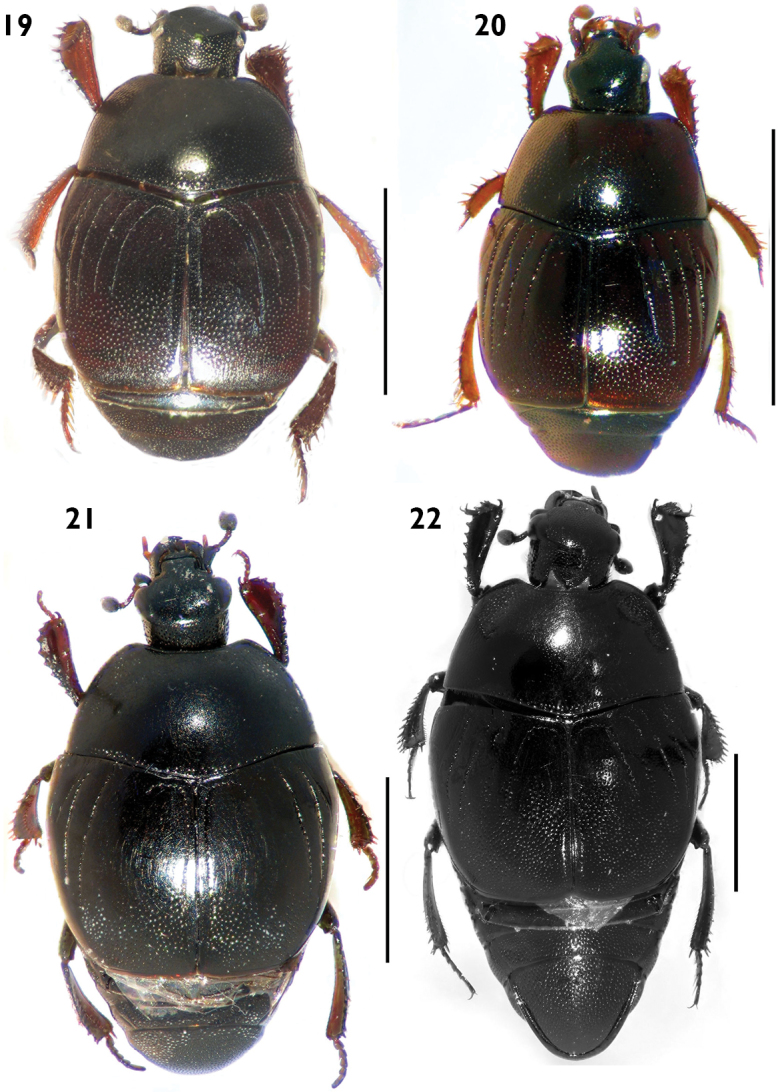
Habitus in dorsal view. **19** *Euspilotus (Hesperosaprinus) modestus* **20** *Euspilotus (Hesperosaprinus) parenthesis* **21** *Euspilotus (Hesperosaprinus) connectens* **22** *Euspilotus (Hesperosaprinus) azureus*. Scale bars: 2 mm.

Specimens were cleaned with water and detergent using a Haier ultrasonic cleaner. Diagnoses were made using a Bausch and Lomb stereomicroscope with magnification between 45× and 60×. Measurements (given in millimeters) were taken with an ocular micrometer. Body length was measured from anterior angle of pronotum to elytral apex, without including head and abdominal terga (propygidium and pygidium) and defined as follows: small 0.5–1.9 mm, medium 2.0–3.9 mm and large 4.0–8.0 mm. Body width was measured at maximum width of elytra, in humeral part. Terminology follows Lackner (2010). The main striae and parts of the body depicted in Figs 1 and 2 were taken from Lackner (2010). Digital photographs of the specimens were taken with a Canon S50 adapted to a Leica MZ6 stereomicroscope. Final images of the specimens (Figs 3–22) were produced with the image stacking freeware CombineZM (Hadley 2006).

## Results

The list of histerids of forensic importance in Argentina comprises 16 species distributed in 13 provinces (Table I). In order to enable a more accurate use of the key, diagnosis of each species with habitus photographs are provided.

### Key to species of Histeridae associated with carcasses in Argentina

**Table:** 

1.	Prosternal lobe present (Fig. 3)	2
1´	Prosternal lobe absent (Fig. 4)	4
2.	Labrum with setae (Fig. 5)	*Carcinops (Carcinops) troglodytes* (Paykull)
2´	Labrum without setae	3
3	Head prognathous, not retractile; mandibles long, prominent, as long as head; pronotum and elytra lacking punctures; length greater than 6.9 mm (Fig. 6)	*Hololepta (Leionota) reichii* Marseul
3´	Head hypognathous, retractile; mandibles short, as long as half of head; pronotum and elytra with finer and sparse punctation; length less than 1.5 mm (Fig. 7)	*Phelister rufinotus* Marseul
4	Pronotal hypomeron setose in dorsal view	5
4´	Pronotal hypomeron glabrous in dorsal view	10
5	Elytronwith five dorsal striae, the fifth between the fourth dorsal and sutural striae (Fig. 8)	*Euspilotus* (sensu stricto) *lacordaire*i (Marseul)
5´	Elytronwith four dorsal striae, fifth stria absent (Figs 9, 10, 13–15)	6
6	Elytron black, lacking spots (Figs 9, 10)	7
6´	Elytronblack with orange, yellow or white spots (Figs 13–15)	8
7	Outer margin of protibiae with teeth much expanded and 6 denticles (Fig. 11); elytron with coarse and dense punctation, with a shining area with finer and sparse punctation between the fourth dorsal and sutural striae, narrowed apically; length greater than 4.4 mm (Fig. 9)	*Euspilotus (sensu stricto) patagonicus* (Blanchard)
7´	Outer margin of protibiae with teeth moderately expanded and 7-8 denticles (Fig. 12); elytronwith very coarse and dense punctation, with a shining impunctate area between the fourth dorsal and sutural striae, wider apically; length less than 2.9 mm (Fig. 10)	*Xerosaprinus (Xerosaprinus) diptychus* (Marseul)
8	Elytral spot with a digitiform projection towards apex (Fig. 13)	*Euspilotus* (sensu stricto) *richteri* Lewis
8´	Elytral spot straight on distal edge (Figs 14–15)	9
9	Elytral spot with two digitiform projections anterad, the outer one close to but not reaching the basal edge (Fig. 14)	*Euspilotus* (sensu stricto) *lepidus* (Erichson)
9´	Elytral spot with three digitiform projections anterad, away from basal edge (Fig. 15)	*Euspilotus* (sensu stricto) *ornatus* (Blanchard)
10	Anterior half of elytronwith very coarse and dense punctation, with a shining impunctate area between the fourth dorsal and sutural striae (Fig. 16)	*Euspilotus (Hesperosaprinus) caesopygus* (Marseul)
10´	Anterior half of elytronwith finer and sparse punctation, lacking shining impunctate areas (Figs 17–22)	11
11	Dorsal elytral striae 3–4 present, well demarcated on anterior half (Figs 17–20)	12
11´	Dorsal elytral striae 3 absent or marked as a row of impressed punctures on basal area, stria 4 present or reduced to a rounded arch basally connected to the sutural stria (Figs 21–22)	15
12	Pronotum with a single fovea on each side close to anterior angles or with a longitudinal lateral depression on each side close to lateral margins with coarse and dense punctation (Figs 17–18)	13
12´	Pronotum lacking fovea or longitudinal lateral depression (Figs 19–20)	14
13	Pronotum with a single depression on each side close to anterior angles, with coarse and dense punctation; distal half of elytra, propygidium and pygidium with ocellate punctation, a small puncture within a large puncture (Fig. 17)	*Euspilotus (Hesperosaprinus) strobeli* (Steinheil)
13´	Pronotum with a longitudinal lateral depression on each side, with coarse and dense punctation; distal half of elytra, propygidium and pygidium with regular punctation (Fig. 18)	*Euspilotus (Hesperosaprinus) pavidus* (Erichson)
14	Elytron with inner subhumeral stria; length greater than 2.5 mm (Fig. 19)	*Euspilotus (Hesperosaprinus) modestus* (Erichson)
14´	Elytron lacking inner subhumeral stria; length less than 2.2 mm (Fig. 20)	*Euspilotus (Hesperosaprinus) parenthes*is (Schmidt)
15	Pronotum with marginal stria away from lateral margin; pygidium with a transverse subapical groove not reaching lateral margins (Fig. 21)	*Euspilotus (Hesperosaprinus) connecte*ns (Paykull)
15´	Pronotum with marginal stria very close to lateral margin; pygidium with a transverse subapical groove reaching lateral margins (Fig. 22)	*Euspilotus (Hesperosaprinus) azure*us (Salberg)

### Diagnoses of species

#### Carcinops ( Carcinops) troglodytes

(Paykull)

http://species-id.net/wiki/Carcinops_troglodytes

Figures 3 5

##### Diagnosis.

Small size (length: 2.1–2.3 mm, width: 1.4–1.6 mm). Body oval, elongated, parallel, black, shiny, with reddish legs. Pronotum with finer and sparse punctation, longer on lateral area, with a large puncture on medial part close to posterior margin. Pronotal hypomeron glabrous in dorsal view. Elytron with finer and sparse punctation in intervals; dorsal elytral striae 1–5 complete, well demarcated with punctures, sutural stria present, reduced on basal part. Pygidium without grooves. Protibiae with teeth expanded and 2 short, separated denticles and a long apical spur; proximal half of outer margin serrate, with small spurs.

##### Distribution.

Cosmopolitan (Mazur 2011). **New record for Argentina**.

#### Hololepta ( Leionota) reichii

Marseul

http://species-id.net/wiki/Hololepta_reichii

Figure 6

##### Diagnosis.

Large size (length: 6.9 mm, width: 5.3 mm). Body black, shiny, depressed, elongated, parallel, head prognathous, not retractile, mandibles long, prominent, as long as head. Pronotum lacking punctures, with marginal stria well demarcated, in males ending in a fovea on anterior angles. Pronotal hypomeron glabrous in dorsal view. Elytron lacking spot and punctures, with only two dorsal striae, first stria reduced to anterior half, second complete, almost reaching apex. Propygidium larger than pygidium, pygidium without grooves. Protibiae with four teeth, the two distal ones longer.

##### Distribution.

Argentina, Brazil, French Guiana, Mexico and Central America (Mazur 1984, 2011).

#### Phelister rufinotus

Marseul

http://species-id.net/wiki/Phelister_rufinotus

Figure 7

##### Diagnosis.

Small size (length: 1.5 mm, width: 1.3 mm). Body oval, black, shiny, with elytron reddish or black rufescent. Pronotum with finer and sparse punctation, larger on medial part close to posterior margin. Pronotal hypomeron glabrous in dorsal view. Elytron with finer and sparse punctation in intervals; dorsal elytral striae 1–4 complete, fifth present on distal half and with a large basal puncture; sutural stria present on distal half. Pygidium with finer and dense punctation and without grooves. Protibiae with outer margin not expanded and with 7 separated denticles.

##### Distribution.

Brazil (Mazur 2011). **New record for Argentina**.

#### Xerosaprinus ( Xerosaprinus) diptychus

(Marseul)

http://species-id.net/wiki/Xerosaprinus_diptychus

Figures 10 12

##### Diagnosis.

Smallto medium size (length: 1.8–2.9 mm, width: 1.7–2.4 mm). Body oval, black to dark brown, shiny. Pronotum with coarse and dense punctation on anterior, lateral and basal areas, disc small, with finer and sparse punctation. Pronotal hypomeron setose in dorsal view. Elytron with coarse and very dense punctation seemingly rugose on distal half and on proximal half in intervals 1–3, with a smooth, shining area between the fourth dorsal stria, the sutural stria and the rounded arch; elytral dorsal striae 1–4 complete on anterior half, sometimes the first and third vestigial, fourth and sutural striae connected by a rounded arch. Pygidium without grooves. Protibiae with teeth moderately expanded and 7–8 denticles (Fig. 12).

##### Distribution.

Mexico (Mazur 2011) and Argentina (Aballay et al. 2008, 2012).

#### Euspilotus ( sensu stricto) lacordairei

(Marseul)

http://species-id.net/wiki/Euspilotus_lacordairei

Figure 8

##### Diagnosis.

Medium to large size (length: 3.4–4.5 mm, width: 3.3–3.8 mm). Body black reddish. Pronotum with coarse and dense punctation on anterior, lateral and basal areas, disc small, with finer and sparse punctation. Pronotal hypomeron setose in dorsal view. Elytron with coarse and dense punctation on distal half, projecting anterad in intervals 1-3, shortest in interval 4; with five dorsal striae well demarcated, 1–4 complete on anterior half, fifth reduced between the fourth dorsal and sutural striae, fourth and sutural striae connected by a rounded arch. Pygidium without grooves. Protibiae with expanded outer margin and 10–11 short, reddish denticles.

##### Distribution.

Argentina, Bolivia and Chile (Mazur 2011; Aballay et al. 2008, 2012).

#### Euspilotus ( sensu stricto) patagonicus

(Blanchard)

http://species-id.net/wiki/Euspilotus_patagonicus

Figures 9 11

##### Diagnosis.

Large size (length: 4.4–5.8 mm, width: 3.9–4.7 mm). Body black. Pronotum with large, shiny disc, with finer and sparse punctation, lateral and basal areas with coarse and dense punctation, with a punctate depressed area on anterior angles, without punctures behind anterior margin. Pronotal hypomeron setose in dorsal view. Elytronwith coarse and dense punctation on distal half, projecting towards anterior half in intervals 1-4, not reaching inner subhumeral stria, the basal area of fourth and sutural striae, with a shining area with finer and sparse punctation between the fourth dorsal and sutural striae, narrowed apically; elytral dorsal striae 1–4 complete on anterior half, sutural stria sometimes absent in(on) basal part. Pygidium without grooves. Protibiae with teeth much expanded and 5–6 short denticles wider on base (Fig. 11).

##### Distribution.

Argentina, Bolivia and Chile (Mazur 2011).

#### Euspilotus ( sensu stricto) richteri

Lewis

http://species-id.net/wiki/Euspilotus_richteri

Figure 13

##### Diagnosis.

Medium size (length: 2.3–3.8 mm, width: 2.1–3.4 mm). Body black, elytron with yellow or white spot. Pronotum with finer and sparse punctation, with a longitudinal lateral area on each side with coarse and dense punctation reaching the marginal stria, with two rows of large punctures on base. Pronotal hypomeron setose in dorsal view. Elytron with punctation coarse and dense on posterior half, finer and sparser on anterior half between intervals 2, 3 and 4; elytral dorsal striae 1–4 complete on anterior third, third stria sometimes reduced in basal area, fourth and sutural striae connected by a rounded arch; elytral spot with a digitiform projection towards apex, with two digitiform projections anterad, the outer one between the first and third dorsal elytral striae, the inner one between the fourth dorsal and sutural striae, sometimes between anterior margin, first and fourth dorsal striae and humerus with small yellow spots, making the anterior margin of the large elytral spot fuzzy. Pygidium: female with subapical groove V-shaped, male without grooves. Protibiae with outer margin expanded and 11–12 short, reddish denticles.

##### Distribution.

Argentina, Chile and Paraguay (Mazur 2011).

#### Euspilotus ( sensu stricto) lepidus

(Erichson)

http://species-id.net/wiki/Euspilotus_lepidus

Figure 14

##### Diagnosis.

Medium size (length: 2.3–3.3 mm, width: 1.86–2.3 mm). Body black, elytron with yellow or white spot. Pronotum with finer and sparse punctation, with a shining area on disc, with a longitudinal lateral area on each side with coarse and dense punctation, with two rows of large punctures on base. Pronotal hypomeron setose in dorsal view. Elytron with punctation coarse and dense on posterior half, finer and sparser on anterior half defining a shining area between intervals 2, 3 and 4; elytral dorsal striae 1, 2 and 4 complete on anterior half, third stria reduced to a short row of punctures on basal area, fourth and sutural striae connected by a rounded arch; elytral spot with distal margin straight and two digitiform projections anterad, the outer one between the first and second (or third) dorsal striae, the inner one towards the fourth dorsal elytral stria. Pygidium without grooves. Protibiae with outer margin expanded and 10–13 denticles.

##### Distribution.

Argentina, Bolivia, Chile, and Peru (Mazur 2011).

#### Euspilotus ( sensu stricto) ornatus

(Blanchard)

http://species-id.net/wiki/Euspilotus_ornatus

Figure 15

##### Diagnosis.

Medium size (length: 2.5–3.5 mm, width: 2.3–3.2 mm). Body black, elytron with yellow or orange spot. Pronotum: disc with finer and sparse punctation, lateral areas and base with coarse and dense punctation. Pronotal hypomeron setose in dorsal view. Elytron with punctation coarse and dense on posterior half, finer and sparser on anterior half defining a shining area between intervals 3 and 4; elytral dorsal striae 1–2 and 4 complete on anterior half, third interrupted, fourth and sutural striae connected by a rounded arch; elytral spot occupying the distal half of elytron with distal margin straight and three digitiform projections anterad, the outer one between the first and second dorsal striae, the medial one between the third and fourth dorsal elytral striae, and the inner one close to the sutural elytral stria. Pygidium without grooves. Protibiae with outer margin expanded and 8–10 short, reddish denticles.

##### Distribution.

Argentina and Chile (Mazur 2011; Aballay et al. 2008, 2012).

#### Euspilotus ( Hesperosaprinus) caesopygus

(Marseul)

http://species-id.net/wiki/Euspilotus_caesopygus

Figure 16

##### Diagnosis.

Medium to large size (length: 3.2–4.3 mm, width: 2.7–3.7 mm). Body black. Pronotum with coarse and dense punctation, disc small, with finer and sparse punctation. Pronotal hypomeron glabrous in dorsal view. Elytron with coarse and very dense punctation seemingly rugose, dorsal elytral striae 1–4 absent or vestigial, sutural stria present, lacking rounded arch, with a shining area on anterior half between the fourth dorsal and sutural striae which presents a finer and sparse punctation visible only at 60× magnification. Pygidium: female with subapical groove, male without grooves. Protibiae with outer margin expanded and 10 short, reddish denticles.

##### Distribution.

Argentina and Bolivia (Mazur 2011).

#### Euspilotus ( Hesperosaprinus) strobeli

(Steinheil)

http://species-id.net/wiki/Euspilotus_strobeli

Figure 17

##### Diagnosis.

Medium to large size (length: 3.5–4.0 mm, width: 2.9–3.9 mm). Body black to metallic blue. Pronotum with a large, shiny disc with finer and sparse punctation, with coarse and dense punctation on lateral area and in a single depression on each side close to anterior angles. Pronotal hypomeron glabrous in dorsal view. Elytron with finer and sparse punctation in the intervals on proximal half; distal half with coarse and dense ocellate punctation, a small puncture within a large puncture, with a smooth, shining area between the fourth dorsal and sutural striae; elytral striae 1-2 almost complete, 3-4 reduced but surpassing the middle of elytron on posterior half, fourth dorsal and sutural striae connected by a rounded arch, lacking inner subhumeral stria. Pygidium with ocellate punctation and with a complete subapical groove in the middle with internal ramifications. Protibiae with outer margin expanded and 7–8 short, reddish denticles.

##### Distribution.

Argentina and South Brazil (Mazur 2011).

#### Euspilotus ( Hesperosaprinus) pavidus

(Erichson)

http://species-id.net/wiki/Euspilotus_pavidus

Figure 18

##### Diagnosis.

Medium size (length: 2.4–3.8 mm, width: 2.1–3.2 mm). Body black with elytron dark reddish. Pronotum with large, shiny, and smooth disc with finer and sparse punctation; anterior, lateral and basal areas with coarse and dense punctation, with two longitudinal, lateral, depressed punctate areas. Pronotal hypomeron glabrous in dorsal view. Elytron with coarse and dense punctuation on distal third from interval 2 to sutural stria, on proximal half with finer and sparse punctation in intervals 1-4; elytral striae 1-2 almost complete, second longer than first, 3-4 surpassing the middle of elytron on posterior half, with inner subhumeral stria well demarcated, sometimes reduced. Pygidium with punctures, without grooves. Protibiae with outer margin expanded and 7–8 short, reddish denticles.

##### Distribution.

Argentina, Bolivia, Brazil, French Guiana, Paraguay, Uruguay, Suriname, and Central America (Arriagada 1987; Mazur 2011; Aballay et al. 2008, 2012).

#### Euspilotus ( Hesperosaprinus) modestus

(Erichson

http://species-id.net/wiki/Euspilotus_modestus

Figures 4 19

##### Diagnosis.

Medium to large size (length: 2.5–4.0 mm, width: 2.4–2.7 mm). Body black to reddish. Pronotum with fine and sparse punctation on disc, larger and deeper on lateral area. Pronotal hypomeron glabrous in dorsal view. Elytron with finer and sparse punctation in the intervals on proximal half; distal half with coarse and dense punctation, apically the punctures form elongate wrinkles; elytral striae 1-4 well demarcated, 1-2 surpassing the middle of elytron on posterior half, 3-4 reduced to anterior half; with inner subhumeral stria well demarcated. Pygidium with coarse and dense punctation, with two short transverse grooves or two longitudinal depressions. Protibiae with outer margin expanded and 8–9 short, reddish denticles, the basal fourth very small.

##### Distribution.

Argentina, Brazil, French Guiana, Paraguay, Uruguay and Venezuela (Mazur 2011; Aballay et al. 2008, 2012).

#### Euspilotus ( Hesperosaprinus) parenthesis

(Schmidt)

http://species-id.net/wiki/Euspilotus_parenthesis

Figure 20

##### Diagnosis.

Smallto medium size (length: 1.7–2.2 mm, width: 1.3–1.8 mm). Body black reddish. Pronotum with fine and sparse punctation over the whole surface area, larger on lateral area. Pronotal hypomeron glabrous in dorsal view. Elytron with coarse and dense punctation on distal half, with finer and sparse punctation in the intervals on proximal half; elytral striae 1-2 almost complete, 3-4 reduced but surpassing the middle of elytron on posterior half; lacking inner subhumeral stria. Pygidium with coarse and dense punctation with or without a short subapical groove, if present it is parenthesis-shaped and concave anterad, not reaching lateral margin of pygidium. Protibiae with outer margin expanded and 7–8 short, reddish denticles.

##### Distribution.

Brazil (Mazur 2011) and Argentina (Aballay et al. 2008, 2012).

#### Euspilotus ( Hesperosaprinus) connectens

(Paykull)

http://species-id.net/wiki/Euspilotus_connectens

Figure 21

##### Diagnosis.

Medium to large size (length: 2.6–3.8 mm, width: 2.2–3.2 mm). Body black. Pronotum with a large, shiny disc with finer and sparse punctation, with coarse and dense punctation on lateral and basal areas and in a single rounded, shallow depression on each side close to anterior angles; with marginal stria away from lateral margin. Pronotal hypomeron glabrous in dorsal view. Elytron with proximal 2/3 lacking punctures, distal third with coarse and dense punctation between the second elytral dorsal and sutural striae; elytral dorsal striae 1–2 almost complete, second larger, third absent or reduced to a short row of punctures on basal area, fourth absent or reduced to a short row of punctures on basal area connected by a rounded arch with sutural stria. Pygidium with punctures and with a transverse subapical groove not reaching lateral margins. Protibiae with outer margin expanded and 7–8 short, reddish denticles.

##### Distribution.

Argentina, Brazil and Uruguay (Mazur 2011).

#### Euspilotus ( Hesperosaprinus) azureus

(Sahlberg)

http://species-id.net/wiki/Euspilotus_azureus

Figure 22

##### Diagnosis.

Medium to large size (length: 2.9–5.5 mm, width: 2.5–4.7 mm). Body black or metallic blue. Pronotum with a large, shiny disc with finer and dense punctation visible only at 60× magnification, larger on lateral areas and in a single depression on each side close to anterior angles; with marginal stria very close to lateral margin. Pronotal hypomeron glabrous in dorsal view. Elytron with finer and sparse punctation in the intervals on proximal half; distal half with coarse and dense punctation between the second interval and sutural stria; elytral dorsal striae 1–2 almost complete, third absent or reduced to a short row of punctures on basal area, fourth complete on anterior half, fourth and sutural striae connected by a rounded arch. Pygidium with punctures and with a transverse subapical groove reaching lateral margins. Protibiae with outer margin expanded and 7–13 short, reddish denticles, the most basal ones very small.

##### Distribution.

Argentina, Brazil and Venezuela (Mazur 2011).

**Figure 23.**
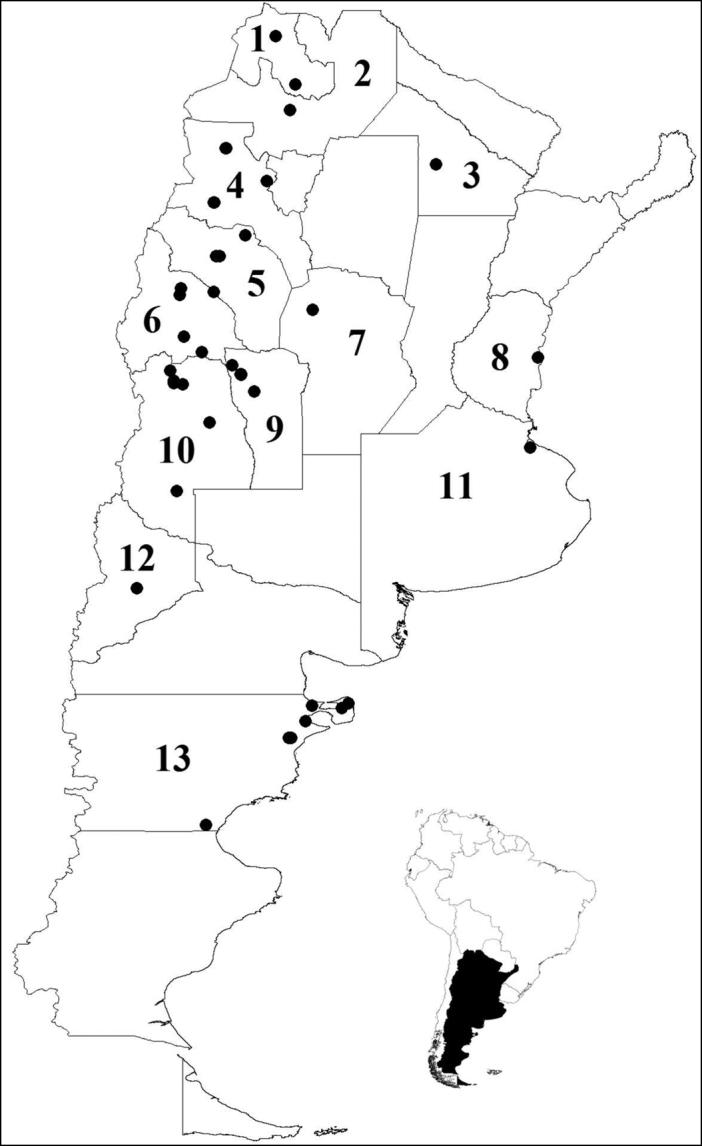
Geographical distribution of sixteen species of Histeridae in Argentina. Provinces: **1** Jujuy: *Euspilotus (Hesperosaprinus) caesopygus*, *Euspilotus* (s. str.) *lacordairei*, *Euspilotus* (s. str.) *lepidus* **2** Salta: *Euspilotus (Hesperosaprinus) caesopygus*, *Euspilotus (Hesperosaprinus) strobeli* **3** Chaco: *Euspilotus* (s. str.) *lacordairei* **4** Catamarca: *Euspilotus (Hesperosaprinus) caesopygus*, *Euspilotus (Hesperosaprinus) pavidus*, *Euspilotus* (s. str.) *lacordairei*, *Euspilotus* (s. str.) *richteri* **5** La Rioja: *Euspilotus (Hesperosaprinus) caesopygus*, *Euspilotus* (s. str.) *lacordairei*, *Euspilotus* (s. str.) *lepidus*, *Euspilotus* (s. str.) *richteri* **6** San Juan**:** *Euspilotus (Hesperosaprinus) modestus*, *Euspilotus (Hesperosaprinus) parenthesis*, *Euspilotus (Hesperosaprinus) pavidus*, *Euspilotus* (s. str.) *lacordairei*, *Euspilotus*. (s. str.) *ornatus*, *Xerosaprinus (Xerosaprinus) diptychus* **7** Córdoba: *Euspilotus (Hesperosaprinus) pavidus* **8** Entre Ríos: *Euspilotus (Hesperosaprinus) pavidus* **9** San Luis: *Euspilotus (Hesperosaprinus) caesopygus*, *Euspilotus (Hesperosaprinus) pavidus*, *Euspilotus* (s. str.) *lacordairei*, *Euspilotus* (s. str.) *ornatus* **10** Mendoza: *Carcinops* (s. str.) *troglodytes*, *Euspilotus (Hesperosaprinus) azureus*, *Euspilotus* (Hesperosaprinus) *caesopygus*, *Euspilotus (Hesperosaprinus) connectens*, *Euspilotus (Hesperosaprinus) modestus*, *Euspilotus (Hesperosaprinus) parenthesis*, *Euspilotus (Hesperosaprinus) pavidus*, *Euspilotus (Hesperosaprinus) strobeli*, *Euspilotus* (s. str.) *lacordairei*, *Euspilotus*(s. str.) *lepidus*, *Euspilotus* (s. str.) *ornatus*, *Euspilotus* (s. str.) *patagonicus*, *Euspilotus* (s. str.) *richteri*, *Hololepta (Leionota) reichii*, *Phelister rufinotus*, *Xerosaprinus diptychus* **11** Buenos Aires: *Euspilotus* (s. str.) *patagonicus* **12** Neuquén: *Euspilotus* (s. str.) *patagonicus* **13** Chubut: *Carcinops* (s. str.) *troglodytes*, *Euspilotus (Hesperosaprinus) modestus*, *Euspilotus* (s. str.) *lacordairei*, *Euspilotus* (s. str.) *ornatus*, *Euspilotus* (s. str.) *patagonicus*, *Euspilotus*(s. str.) *richteri*.

## Discussion

The 16 Histeridae species collected in this study on carcasses in Argentina are grouped into three of the 11 subfamilies: Saprininae (twelve species of *Euspilotus* Lewis and one species of *Xerosaprinus* Wenzel), Histerinae (one species of *Hololepta* Paykull and one species of *Phelister* Marseul) and Dendrophilinae (one species of *Carcinops* Marseul).

Species of *Euspilotus*, *Xerosaprinus* and *Phelister* have been recorded as attracted by carcasses (Kovarik and Catherino 2001), and species of *Hololepta* and *Carcinops* are associated with rotting vegetation, especially cacti and bromeliads (Arriagada 1986, Kovarik and Catherino 2001). The species *Carcinops (Carcinops) troglodytes* has been found to be an effective natural enemy of synanthropic muscoid Diptera: *Musca domestica* Linnaeus (Muscidae) and *Chrysomya putoria* (Wiedemann) (Calliphoridae), and considered a potential biological control agent for the coleopteran *Alphitobius diaperinus* Panzer (Tenebrionidae) that develops in chicken droppings in Brazil (Lopes et al. 2006, Santoro et al. 2010); in Chile it was collected on dry goat’s dung (Arriagada 1986). *Hololepta (Leionota) reichii* has been considered a predator of larvae and pupae of *Melipona compressipes manaosensis* Schwarzand *Melipona seminigra merrillae* Cockerell(Hymenoptera: Apidae)inside bee hives (Coletto-Silva and Freire 2006). In this study, *Carcinops troglodytes* was collected on pig and *Hololepta reichii* on human carcasses, in both cases with presence of Calliphoridae larvae.

Nine species of Histeridae constitute new records from the cadaveric fauna in Argentina: *Euspilotus caesopygus*, *Euspilotus connectens*, *Euspilotus lepidus*, *Euspilotus richteri*, *Euspilotus strobelis*, *Euspilotus azureus*, *Hololepta reichii*, *Phelister rufinotus* and *Carcinops troglodytes*. All of them were collected mostly on human and pig carcasses. The remaining seven species associated with carcasses listed in this key were recorded previously for the country in Buenos Aires (Centeno et al. 2002), Neuquén (Oliva and Ravioli 2004) and San Juan provinces (Aballay et al. 2008, 2012). Two species are new records for Argentina: *Phelister rufinotus* and *Carcinops troglodytes*.

Histeridae of forensic importance were already cited in the literature, for instance in Central Europe adults of *Saprinus planiusculus* Motschulsky and *Saprinus semistriatus* (Scriba) are predictable at a specific time period in the cadaver succession because they have a short period of residency in the carcasses depending on their specialized feeding habits, therefore they are good tools for estimating PMI indicators (Matuszewski et al. 2010).

*Operclipygus hospes* (Lewis) was recorded from Brazil in buried bodies of rabbits in summer and autumn, and it was suggested that this species plays an important role in forensic entomology as a seasonal indicator (Corrêa et al. 2012).

Further research is necessary to establish the specific time period in the cadaver succession for which the species cited in the present article can be predictable and could be used to estimate PMI indicators based on succession patterns. In addition, immature stages can be useful in forensic entomology because they are reared within the body and collected in advanced stages of decomposition (Aballay pers. obs.) but the duration of larval development is variable and depends on the species (Kovarik and Catherino 2005).

Due to the limited information concerning development of larvae of Histeridae species (Kovarik and Catherino 2005), research studies should be conducted on their life cycle and to this end it is essential to achieve a correct identification of the adult necrophilous histerids. In this sense we consider that the present paper is a basic tool for undertaking these studies.

**Table 1.** List of Histeridae species collected on vertebrate carcasses and from baited traps in Argentina and their geographic distribution by provinces. * = baited traps.

Species	N°	Substratum /carcasses	Province	Geographic Coordinates	Altitude (m)	Collector/ reference
*Carcinops (s. str.) troglodytes*	4	Pig	Mendoza	32°53'49.3"S, 68°52'23.9"W	839	Aballay (2012)
*Carcinops (s. str.) troglodytes*	4	Sheep	Chubut	43°16'18.2"S, 65°26'23.3"W	39	Arriagada G.
*Euspilotus (Hesperosaprinus) azureus*	192	Pig	Mendoza	32°53'58.4"S, 68°52'22.1"W	841	Aballay (2012)
*Euspilotus (Hesperosaprinus) caesopygus*	5	Pig	Mendoza	32°53'53.3"S, 68°52'26.2"W	850	Aballay (2012)
*Euspilotus (Hesperosaprinus) caesopygus*	2	Human,	Mendoza	32°32'07.5"S, 68°58'42.8"W	1424	Aballay F. (forensic cases)
*Euspilotus (Hesperosaprinus) caesopygus*	3	Pig	Jujuy	24°09'54.1"S, 65°18'37.7"W	1383	Quiroga N.
*Euspilotus (Hesperosaprinus) caesopygus*	1	Pig	Salta	24°54'40"S, 65°28'16"W	1379	Ayón R.
*Euspilotus (Hesperosaprinus) caesopygus*	121	Squid	La Rioja	29°10'45.3"S, 67°37'33.9"W	1806	Arriagada G.
*Euspilotus (Hesperosaprinus) caesopygus*	4	Squid *	Catamarca	27°36'35.4"S, 67°41'48.5"W	1752	Arriagada G.
*Euspilotus (Hesperosaprinus) caesopygus*	1	Dog	San Luis	32°37'37.8"S, 66°54'35.5"W	744	Arriagada G.
*Euspilotus (Hesperosaprinus) connectens*	12	Pig	Mendoza	32°53'57.6"S, 68°52'32.4"W	847	Aballay (2012)
*Euspilotus (Hesperosaprinus) modestus*	21	Human	Mendoza	32°49'18.4"S, 68°52'38.9"W	788	Aballay F. (forensic cases)
*Euspilotus (Hesperosaprinus) modestus*	4	Cow	San Juan	31°59'51.1"S, 68°03'20.3"W	541	Arriagada G
*Euspilotus (Hesperosaprinus) modestus*	2	Pig	San Juan	31°32'34.1"S, 68°34'38.2"W	673	Aballay et al. (2008, 2012)
*Euspilotus (Hesperosaprinus) modestus*	91	Pig	Mendoza	32°53'53.3"S, 68°52'26.2"W	850	Aballay (2012)
*Euspilotus (Hesperosaprinus) modestus*	91	Sardine*	Chubut	43°16'37.1"S, 65°29'49.8"W	68	Arriagada G.
*Euspilotus (Hesperosaprinus) parenthesis*	2	Pig	San Juan	31°32'34.9"S, 68°34'35.9"W	674	Aballay et al. (2008, 2012)
*Euspilotus (Hesperosaprinus) parenthesis*	30	Pig	Mendoza	32°53'49.3"S, 68°52'23.2"W	850	Aballay (2012)
*Euspilotus (Hesperosaprinus) pavidus*	5	Human	Mendoza	32°56'14.2"S, 68°36'32.9"W	653	Aballay F. (forensic cases)
*Euspilotus (Hesperosaprinus) pavidus*	63	Pig	San Juan	31°32'32.1"S, 68°34'44.8"W	675	Aballay et al. (2008, 2012)
*Euspilotus (Hesperosaprinus) pavidus*	163	Pig	Mendoza	32°53'58.4"S, 68°52'22.1"W	841	Aballay (2012)
*Euspilotus (Hesperosaprinus) pavidus*	70	Donkey	Catamarca	26°59'22.1"S, 66°08'42.1"W	2121	Arriagada G.
*Euspilotus (Hesperosaprinus) pavidus*	15	Horse	San Luis	32°38'43.4"S, 66°53'52.7"W	717	Arriagada G.
*Euspilotus (Hesperosaprinus) pavidus*	45	Chicken *	Córdoba	30°44'39.8"S, 64°48'35.5"W	480	Arriagada G.
*Euspilotus (Hesperosaprinus) pavidus*	100	Cow	Entre Rios	32°08'39.1"S, 58°13'04.3"W	31	Arriagada G.
*Euspilotus (Hesperosaprinus) strobeli*	1	Pig	Salta	24°54'40"S, 65°28'16"W	1379	Ayón R.
*Euspilotus (Hesperosaprinus) strobeli*	1	Cow	Mendoza	34°03'18.1"S, 67°49'13.8"W	537	Flores G.
*Euspilotus (Hesperosaprinus) strobeli *	1	Chicken *	Mendoza	34°03'25.1"S, 67°49'11.8"W	534	Arriagada G
*Euspilotus (s. str.) lacordairei*	25	Pig	San Juan	31°32'34.1"S, 68°34'38.2"W,	673	Aballay et al. (2008, 2012)
*Euspilotus (s. str.) lacordairei*	2	Pig	San Juan	30°07'01.1"S, 68°39'43.9"W	1144	Aballay F.
*Euspilotus (s. str.) lacordairei*	867	Pig	Mendoza	32°53'57.6"S, 68°52'32.2"W	850	Aballay (2012)
*Euspilotus (s. str.) lacordairei *	2	Horse	San Luis	32°38'34.4"S, 66°53'35.7"W	720	Arriagada G.
*Euspilotus (s. str.) lacordairei *	2	Cow	San Luis	32°22'08.2"S, 67°09'37.3"W	556	Aballay F.
*Euspilotus (s. str.) lacordairei *	4080	Sardine*, Squid*	Chubut	43°16'37.1"S, 65°29'49.8"W	68	Arriagada G.
*Euspilotus (s. str.) lacordairei *	20	Rat	Chubut	42°24'11.1"S, 63°57'25.4"W	6	Cheli G
*Euspilotus (s. str.) lacordairei *	2	Lesser rhea	Chubut	42°20'21.8"S, 64°49'11.2"W	50	Flores G;.
*Euspilotus (s. str.) lacordairei *	2	Sheep	Chubut	42°20'28.8"S, 64°49'09.2"W	48	Flores G.
*Euspilotus (s. str.) lacordairei *	5	Vicuña	Jujuy	22°44'52.4"S, 65°53'12.9"W	3667	Arriagada G.
*Euspilotus (s. str.) lacordairei *	166	Squid *	La Rioja	29°10'43.5"S, 67°31'49.9"W	1196	Arriagada G.
*Euspilotus (s. str.) lacordairei *	2	Donkey	Catamarca	26°59'22.1"S, 66°08'42.1"W	2121	Arriagada G.
*Euspilotus (s. str.) lacordairei *	1	Snake	Chaco	26°30'16.3"S, 61°11'15.2"W	124	Arriagada G.
*Euspilotus (s. str.) lepidus*	7	Pig	Jujuy	24°09'54.1"S, 65°18'37.7"W	1383	Quiroga N.
*Euspilotus (s. str.) lepidus*	55	Pig	Mendoza	32°53'49.3"S, 68°52'23.9"W	839	Aballay (2012)
*Euspilotus (s. str.) lepidus*	10	Squid *	La Rioja	28°34'17.9"S, 66°47'07.4"W	812	Arriagada G.
*Euspilotus (s. str.) ornatus*	2	Pig	San Juan	31°32'34.9"S, 68°34'35.9"W	674	Aballay et al. (2008, 2012)
*Euspilotus (s. str.) ornatus*	48	Pig	Mendoza	32°53'58.4"S, 68°52'22.1"W	841	Aballay (2012)
*Euspilotus (s. str.) ornatus*	30	Rat	Chubut	45°49'04.7"S, 67°55'59.6"W	680	Cheli G.
*Euspilotus (s. str.) ornatus*	30	Sardine*	Chubut	43°16'30.1"S, 65°29'26.8"W	66	Arriagada G.
*Euspilotus (s. str.) ornatus*	3	Geoffroy´s cat	San Luis	33°08'07.5"S, 66°30'27.9"W	551	Arriagada G.
*Euspilotus (s. str.) patagonicus*	4	Rat	Chubut	42°47'07.5"S, 65°00'43.8"W	9	Cheli G.
*Euspilotus (s. str.) patagonicus*	4	Guanaco	Mendoza	36°03'27.5"S, 68°47'11.1"W	1684	Flores G., Ruiz Manzanos E.
*Euspilotus (s. str.) patagonicus*	1	Pig	Buenos Aires	34°47'13.2"S, 58°26'33.1"W	17	Centeno et al. (2002)
*Euspilotus (s. str.) patagonicus*	1	Human	Neuquén	38°53'54.8"S, 69°56'54.2"W	962	Oliva and Ravioli (2004)
*Euspilotus (s. str.) richteri*	178	Pig	Catamarca	26°01'38.2"S, 67°20'31.6"W	3595	Aballay (2012)
*Euspilotus (s. str.) richteri*	95	Llama	Catamarca	26°01'33.4"S, 67°20'42.5"W,	3585	Aballay (2012)
*Euspilotus (s. str.) richteri *	85	Squid *	Catamarca	27°36'30.1"S, 67°41'04.7"W	1750	Arriagada G.
*Euspilotus (s. str.) richteri*	8	Pig	Mendoza	32°53'57.6"S, 68°52'32.4"W	847	Aballay (2012)
*Euspilotus (s. str.) richteri *	1	Rat	Chubut	42°16'10.4"S, 63°45'32.2"W	40	Cheli G.
*Euspilotus (s. str.) richteri *	76	Squid *	La Rioja	28°34'17.9"S, 66°47'07.4"W	812	Arriagada G.
*Hololepta (Leionota) reichii*	1	Human	Mendoza	32°56'14.2"S, 68°36'32.9"W	653	Aballay F.(forensic cases)
*Phelister rufinotus*	11	Pig	Mendoza	32°53'53.3"S, 68°52'26.2"W	850	Aballay (2012)
*Xerosaprinus diptychus*	72	Pig	San Juan	31°32'32.1"S, 68°34'44.8"W	675	Aballay et al. (2008, 2012)
*Xerosaprinus diptychus*	2	Horse	San Juan	30°13'52.3"S, 67°42'33.8"W	1261	Aballay F.
*Xerosaprinus diptychus*	2	Fox	San Juan	30°19'01.3"S, 68°41'42.3"W	673	Aballay F.
*Xerosaprinus diptychus*	114	Pig	Mendoza	32°53'49.3"S, 68°52'23.9"W	839	Aballay (2012)

## Supplementary Material

XML Treatment for Carcinops ( Carcinops) troglodytes

XML Treatment for Hololepta ( Leionota) reichii

XML Treatment for Phelister rufinotus

XML Treatment for Xerosaprinus ( Xerosaprinus) diptychus

XML Treatment for Euspilotus ( sensu stricto) lacordairei

XML Treatment for Euspilotus ( sensu stricto) patagonicus

XML Treatment for Euspilotus ( sensu stricto) richteri

XML Treatment for Euspilotus ( sensu stricto) lepidus

XML Treatment for Euspilotus ( sensu stricto) ornatus

XML Treatment for Euspilotus ( Hesperosaprinus) caesopygus

XML Treatment for Euspilotus ( Hesperosaprinus) strobeli

XML Treatment for Euspilotus ( Hesperosaprinus) pavidus

XML Treatment for Euspilotus ( Hesperosaprinus) modestus

XML Treatment for Euspilotus ( Hesperosaprinus) parenthesis

XML Treatment for Euspilotus ( Hesperosaprinus) connectens

XML Treatment for Euspilotus ( Hesperosaprinus) azureus
